# New Appliances and Things Medical

**Published:** 1904-07-09

**Authors:** 


### NEW APPLIANCES AND THINGS MEDICAL.

[We shall be glad to receive, at our Office, 28 & 29 Southampton Street, Strand, London, W.C., from the manufacturers, specimens of all new preparations and appliances which may be brought out from time to time.]

### ROBORAT.

(THE ROBORAT COMPANY, MOULTON STREET, STRANGEWAYS, MANCHESTER.)

A SAMPLE of this preparation has been submitted to us. It is a fine powder, and is easily soluble in milk, water, soup, stock, etc. It can be added to any liquid food, and also to cakes, pastry, etc. One of the claims put forward by the proprietors of Roborat is that it contains fat, and in particular a phosphorated fat, lecithin, the nutritive and therapeutic value of which has been established. From metabolic experiments it is shown to be well assimilated both in children and adults. On account of its freeness from carbohydrates it may be recommended in the dietary of diabetics, and by means of it a "bread" has been produced free from carbohydrates.

